# The impact of weight loss on renal function in individuals with obesity and type 2 diabetes: a comprehensive review

**DOI:** 10.3389/fendo.2024.1320627

**Published:** 2024-01-31

**Authors:** Xuemei Gong, Xiaoxi Zeng, Ping Fu

**Affiliations:** ^1^ Department of Nephrology, West China Hospital, Sichuan University, Chengdu, China; ^2^ Division of Nephrology, Kidney Research Institute, West China Hospital, Sichuan University, Chengdu, China; ^3^ West China Biomedical Big Data Center, West China Hospital, Sichuan University, Chengdu, China

**Keywords:** weight loss, obesity, type 2 diabetes, renal function, chronic kidney disease

## Abstract

Obesity and Type 2 Diabetes (T2D) are two highly prevalent diseases that exhibit a complex interplay between them. Obesity serves as a primary risk factor for the development of T2D, and conversely, individuals with T2D often exhibit comorbid obesity. Renal dysfunction emerges as a critical consequence of the convergence of obesity and Type 2 Diabetes, contributing significantly to the overall burden of complications associated with these conditions. Recognizing the profound implications of renal dysfunction in individuals contending with both obesity and Type 2 Diabetes, interventions targeting weight loss have gained prominence as potential therapeutic avenues. Weight loss not only addresses the primary risk factor of obesity but also holds the promise of mitigating the progression of Type 2 Diabetes and its associated renal complications. This comprehensive review aims to explore the impact of weight loss on renal function in individuals contending with the convergence of obesity and T2D.

## 1 Association between obesity, type 2 diabetes, and chronic kidney disease

The global prevalence of obesity and T2D has reached alarming levels. As of 2021, approximately 529 million individuals were living with diabetes globally, with a standardized prevalence rate of 6.1% (5.8–6.5%) (1). Concurrently, obesity’s prevalence and severity continue to rise within the T2D and general populations. Startling statistics from the “National Diabetes Statistics Report (2017)” indicate that an overwhelming 87.5% of adult diabetic patients are either overweight or obese (2).

Obesity is unequivocally established as a critical risk factor for various diseases, including diabetes, hypertension, and cardiovascular disorders (3), all of which have significant adverse effects on kidney health, leading to conditions such as diabetic kidney disease (DKD) (4, 5) and end-stage renal disease (ESRD) (6). A study by Kamel et al. found that the risk of DKD among individuals with diabetes rises with increasing BMI, ranging from 0.91 for overweight individuals (BMI > 25 kg/m^2^) to 2.16 for severely obese individuals (BMI > 40 kg/m^2^) (7). These effects are mediated by several factors, including alterations in glomerular hemodynamics, heightened sympathetic nervous activity, hypertension, chronic inflammation, endothelial dysfunction, and so on (8). Intriguingly, even in the absence of diabetes, obese individuals may experience a heightened frequency and severity of proteinuria, with obesity independently contributing to the development of glomerulopathy. Obesity also negatively impacts key Chronic kidney disease(CKD)-associated risk factors, encompassing blood lipid levels, blood pressure, blood glucose control, and the promotion of insulin resistance (9). Notably, CKD tends to be more prevalent and progresses more rapidly in obese individuals with T2D compared to those with normal weight (2).

Given the intricate intertwining of obesity, T2D, and CKD, weight loss interventions have emerged as prospective strategies to enhance renal function and overall health in patients grappling with obesity and T2D (10). Extensive researches underscored the profound benefits of weight loss on kidney health (11–16). Rebecca O’Brien et al. discovered that among patients aged 19–79 with T2DM who underwent bariatric surgery, bariatric surgery was associated with a lower cumulative incidence of diabetic nephropathy(DN) at 5 years (17). Weight loss interventions, including lifestyle modifications, pharmaceutical interventions, and bariatric surgery, have the potential to enhance insulin sensitivity, regulate glucose levels, and control blood pressure (18, 19). Additionally, weight loss has demonstrated efficacy in reducing oxidative stress and inflammation, key factors in kidney injury development (20).

This review aims to provide a comprehensive analysis of the effects of weight loss interventions on renal function in patients with obesity and T2D. By examining the existing evidence, we seek to contribute valuable insights into the potential of weight loss strategies as renoprotective measures and to advocate for the adoption of personalized and multidisciplinary approaches in managing obesity, T2D, and CKD.

## 2 Obesity and diabetes: primary mechanisms leading to renal dysfunction

### 2.1 Diabetic kidney disease

Hyperglycemia-induced metabolic dysregulation is a widely recognized primary contributor to the onset and progression of DKD (21). The pathophysiology of DKD is multifaceted, encompassing hemodynamic (RAAS activation, endothelial dysfunction), metabolic (accumulation of advanced glycation end-products), pro-inflammatory (reactive oxygen species generation, tumor necrosis factor-α activation), and pro-fibrotic (stimulation of transforming growth factor-β signaling), but in reality these elements interact locally and systemically to form a complex and dynamic interplay resulting in functional and structural changes to the kidney (22). The Renin-Angiotensin-Aldosterone System (RAAS) plays a pivotal role in the pathogenesis of DKD, partly through its promotion of efferent arteriolar constriction and intraglomerular hypertension, as well as its activation of inflammatory and fibrotic pathways (23). Furthermore, factors such as Protein Kinase C-β, oxidative stress mediators, Advanced Glycation End-products (AGE), as well as various cytokines and chemokines, play significant roles in driving DKD progression in the context of hyperglycemic conditions (21, 22).

Obesity, which often co-occurs with diabetes, poses a substantial risk to renal function (24, 25). Accumulation of visceral fat triggers an adipocyte stress response and pro-inflammatory signaling, leading to metabolic dysregulation. Ectopic lipid deposition within the kidneys and the presence of intracellular lipid metabolites, such as ceramides, contribute to oxidative stress and podocyte insulin resistance, resulting in impairment of the glomerular barrier (26). Obesity also exacerbates conditions like hypertension, insulin resistance, Type 2 diabetes, and atherosclerosis, all of which contribute to renal injury and endothelial dysfunction (24, 26). Recent research indicates that obesity may exert an influence on the composition of gut microbiota, potentially impacting the development of diabetic kidney disease (27). Alterations in gut microbiota associated with obesity, including reduced microbial diversity and shifts in microbial species composition, have the potential to compromise gut barrier integrity and promote inflammation, ultimately affecting renal health (28).

### 2.2 Obesity-related glomerulopathy

Recent studies identify obesity as an independent risk factor for renal dysfunction, apart from diabetes and hypertension (29). ORG has emerged as a distinctive subtype of CKD, characterized by proteinuria, enlarged glomeruli, and a gradual decline in renal function (30). Obesity-induced alterations in adipose tissue impact renal health through diverse mechanisms. Adipose tissue stress affects adipokine secretion, altering the adiponectin-to-leptin ratio, associated with renal impairment (26). Obesity also impacts renal sodium handling and hypertension development, with elevated leptin levels stimulating the sympathetic nervous system and activating the renin-angiotensin system (26, 30). Mechanical effects of adipose tissue deposition, like perirenal and renal sinus fat accumulation, may also contribute to hypertension and renal injury (31). These changes may slow peritubular capillary blood flow, promote sodium retention, and lead to chronic renal function decline.

The systemic and local disruptions observed in ORG exhibit similarities to the pathogenesis of DKD. These two diseases interact with each other, leading to progressive renal damage.

## 3 Impact of weight loss on renal function in diabetic patients

Numerous high-quality clinical studies have provided substantial evidence supporting the role of weight loss in alleviating the progression of existing DKD and reducing the long-term incidence of diabetes-related renal complications. As previously discussed, O’Brien et al. conducted a retrospective observational cohort study of 2,205 type 2 diabetes patients who underwent metabolic surgery. A comparison with 11,059 matched non-surgical control subjects revealed a remarkable reduction in the incidence of kidney disease over a 6-year follow-up period. Specifically, metabolic surgery demonstrated a 6.4% incidence in surgical patients compared to 14% in non-surgical controls, with an adjusted hazard ratio of 0.45 [95% CI 0.29-0.71] (17). A matched cohort study by Madsen et al. showed that RYGB surgery led to a significant reduction in T2DM microvascular complications (HR 0.53 [95% CI 0.38–0.73]) and the incidence of type 2 DKD(incidence rate ratio 0.54 [95%CI 0.31–0.94]) (32). Additionally, Glucagon-Like Peptide-1(GLP-1), an incretin hormone, induces weight loss by reducing appetite, delaying gastric emptying, and optimizing insulin and glucagon secretion timing. Studies have demonstrated that Liraglutide not only leads to weight loss but also improves blood sugar control, enhances various cardiovascular markers, and reduces albuminuria. The LEADER RCT (33) confirmed that Liraglutide can slow the occurrence and progression of DKD and alleviate the recurrence of type 2 diabetes after metabolic surgery. Therefore, the use of Liraglutide after metabolic surgery may provide additional renal protective benefits (34). Some medications may potentiate weight loss but are not FDA approved for obesity. Sodium glucose co-transporter 2 inhibitors (SGLT2 inhibitors) also have significant weight-reducing effects. Although their primary purpose is to lower blood sugar by inhibiting renal sodium-glucose co-transporter-2, rather than being a weight loss drug, it has been observed that these agents can substantially improve body weight and exert a potent protective effect on kidney function. A comprehensive meta-analysis has underscored the multifaceted benefits of SGLT-2 inhibitors in patients with T2DM and CKD. These inhibitors not only lowered glycated hemoglobin (–0.29%, 95% CI –0.39 to –0.19) but also demonstrated reductions in blood pressure, body weight, and albuminuria. Furthermore, they mitigated the annual decline in eGFR slope (placebo-subtracted difference of 1.35 mL/1.73 m^2^/year, 95% CI 0.78-1.93) and decreased the risk of the composite renal outcome, encompassing doubling of serum creatinine, end-stage kidney disease, or renal death (HR 0.71, 95% CI 0.53-0.95) (35).

### 3.1 Weight loss interventions primarily alleviate renal damage by improving the following conditions

#### 3.1.1 Diabetes emission

As mentioned earlier, elevated blood glucose levels are a crucial factor in the progression of diabetes patients to diabetic kidney disease. Numerous studies have demonstrated that weight loss interventions can significantly enhance insulin sensitivity, thereby improving blood sugar control and alleviating kidney damage (36). Evidence indicates that early weight loss surgical interventions in T2DM patients can lead to diabetes remission and substantially reduce the residual complications of the disease (36). A notable 10-year follow-up study revealed that 37.5% of patients who underwent surgical treatment sustained diabetes remission throughout the decade. This rate contrasts with 5.5% for medical therapy, 50.0% for biliopancreatic diversion (BPD), and 25.0% for Roux-en-Y gastric bypass (RYGB) (37). Mechanistic research suggests that weight loss primarily reduces intra-abdominal, intramyocellular, and intrahepatocellular lipids under hypocaloric conditions, rather than generalized body fat (38). This loss reverses crucial pathophysiological processes in diabetes, such as enhancing peripheral insulin sensitivity, improving cellular insulin signal transduction, boosting insulin secretion, and decreasing hepatic glucose production (38). The resulting improved insulin sensitivity optimizes glycemic control, thereby preserving glomerular integrity and function, reducing oxidative stress and inflammation, and preserving renal unit structure (39).

#### 3.1.2 Reducing proteinuria and improving kidney structure

Weight loss interventions, particularly surgical procedures, demonstrate sustained remission of albuminuria post-surgery, with significant reductions in albuminuria observed across all baseline albumin-creatinine ratio tertiles, with remission occurring in 78% of patients and parallel studies in Zucker diabetic fatty rats revealed that weight loss and improvements in glycemia following RYGB surgery were accompanied by the normalization of glomerular tuft size, reduced podocyte expression of desmin, and preservation of podocyte foot process morphology (40). Notably, RYGB attenuated podocyte stress and dedifferentiation in the ZDF rat model, likely due to enhanced metabolic control, especially its potent glucose-lowering effect post-surgery (40). Similarly, researchers observed a reduction in urinary protein and the restoration of glomerular injury, attributed to improved glomerular filtration membrane ultrastructure and increased nephrin protein expression (41). Additionally, studies in obese type 2 diabetic patients revealed that calorie restriction also improved glomerular hyperfiltration and various cardiovascular risk factors, while also reducing serum angiotensin II levels, suggesting reduced RAS activity (14). Furthermore, Glucagon-Like Peptide-1 Receptor Agonists (GLP1-RA) like liraglutide can inhibit sodium-hydrogen exchanger 3 in proximal tubular cells, increasing natriuresis and diuresis (42).

#### 3.1.3 Alleviating inflammatory status

Chronic low-grade inflammation is a hallmark of diabetic nephropathy and obesity (43). Researchers have uncovered that the reduction in body weight observed in RYGB rats is associated with a diminished fibrotic Transforming Growth Factor β (TGFβ) signal (44). TGFβ is recognized as a major driver of fibrosis and a key mediator of the hypertrophic and prosclerotic changes in diabetic nephropathy. Consequently, its downregulation appears to be a potential favorable effect in renal damage in patients with obesity and type 2 diabetes (45). Similarly, Bariatric surgery in mouse models activated Peroxisome Proliferator-Activated Receptor Alpha (PPARα), leading to the inhibition of Reactive Oxygen Species (ROS) generation. This action mitigates oxidative stress and reduces renal apoptosis, showcasing the protective effect of bariatric surgery on kidneys affected by diabetic nephropathy (46). Furthermore, Dietary intervention in mouse models attenuate progressive urinary protein excretion and renal inflammation, suggesting that adiposity drives renal inflammation in DKD (47–51). Reduced expression levels of inflammatory factors such as C-Reactive Protein (CRP) and C-C Motif Chemokine Ligand 2 (CCL2) enhance kidney function, decrease renal fibrosis, and improve inflammation control (52). Furthermore, GLP1-RA have been shown to stimulate pathways that reduce reactive oxygen species in the kidneys. Additionally, they contribute to lowering inflammation by decreasing cytokine production and immune cell infiltration (42). This evidence collectively highlights the intricate interplay between inflammation, weight loss interventions, and the potential protective effects on renal health in the context of diabetic nephropathy and obesity.

#### 3.1.4 Gut microbiota changes

Alterations in gut microbiota following weight loss have been associated with improvements in energy balance, enhanced intestinal insulin release, and weight reduction. Recent meta-analyses have revealed significant alterations in gut microbiota and microbial metabolites following weight loss surgery. These changes are closely associated with improved glucose homeostasis, weight reduction, and modifications in gastrointestinal intake and exercise behaviors (53). A detailed investigation focused on diabetic patients undergoing metabolic surgery has unveiled intricate connections among serum metabolomics, gut microbiota composition, and hormonal profiles, particularly concerning improvements in diabetes and metabolic syndrome. Noteworthy correlations between specific gut bacteria, such as the Eubacterium eligens group, and metabolites like lacosamide glucuronide and UDP-L-arabinose were identified. Positive correlations were observed between Enterococcus and metabolites like glutamic acid and vindoline (54). Furthermore, research has shown that medical treatments also significantly alter intestinal microbiota, with observable changes like increased Proteobacteria and variable Bacteroidetes. These shifts correlate with changes in patient weight, and glucose metabolism (55). Additionally, the dysbiotic state induced by a high-fat diet was ameliorated by transitioning to a lower-fat, higher-fiber control diet, especially when combined with sleeve gastrectomy. This led to increased microbial diversity and shifting relative abundances (56). Moreover, the role of short-chain fatty acids (SCFAs), generated through bacterial fermentation of dietary fiber in the colon, has been crucial in protecting mice against the clinical and histologic manifestations of diabetic nephropathy by activating G Protein-Coupled Receptors GPR43 and GPR109 (57). Targeted dietary fiber supplementation to increase SCFA levels has also been found to contribute to better improvements in hemoglobin A1c levels, partly attributed to increased production of glucagon-like peptide-1 (GLP-1) (58). Hence, alterations in gut microbiota composition following weight loss may contribute to improved blood glucose and blood pressure control by enhancing colonic L cell secretion of GLP-1.

## 4 Impact of weight reduction on renal function in obese patients

A growing body of evidence suggests that weight reduction yields positive outcomes in non-diabetic patients with obesity, Several longitudinal studies, including a large cohort study (59), observed a substantial decrease in the risk of CKD in obese patients after weight reduction surgery. Notably, improvements in CKD risk categories are observed at 1 and 7 years after surgery, particularly in patients with moderate to high baseline CKD risk. It demonstrated similarities in the effects of weight reduction on renal function in both diabetic and non-diabetic obese patients. These effects encompass reduced adipose tissue, improved lipid metabolism, and ameliorated inflammatory states.

### 4.1 The benefit of adipose tissue reduction

Post-weight loss, there is a notable decrease in visceral adipose tissue and renal sinus fat. This reduction contributes to a favorable adipokine profile, diminished inflammatory cytokines, and lowered ROS production, potentially resulting in reduced RAAS activation levels (60). Obesity-induced metabolic syndrome disrupts lipid metabolism, leading to abnormal lipid profiles. Weight loss interventions have consistently proven effective in decreasing blood lipid levels and fat deposition, while increasing adiponectin levels, which play a key role in metabolic regulation (15, 61). Adiponectin plays a multifaceted role in metabolic control, promoting fatty acid oxidation through AMP-activated protein kinase (AMPK) activation. AMPK activation enhances insulin sensitivity in crucial insulin-target tissues like skeletal muscle and white adipose tissue, vital for blood glucose control. By stimulating fatty acid oxidation and ceramidase activity, adiponectin counteracts lipotoxicity and oxidative stress (62). Moreover, both intensive lifestyle interventions and bariatric surgery-induced weight loss have been found to reduce lipid accumulation and decrease local and systemic inflammatory microenvironments (60). Additionally, beyond bodyweight reduction, the improvement of the metabolic kidney milieu and restoration of endothelial function could also contribute to the renoprotective effects of regular exercise in chronic diabetic diseases (63). Furthermore, several longitudinal studies exploring the reduction of ectopic renal fat post-weight loss surgery have demonstrated a correlation with improved renal function. Renal sinus fat (RSF), a fat depot at the hilum of the kidney, has been studied for its association with hypertension, and its reduction post-weight loss surgery has been observed. In comparison to the lean control group, obese patients accumulated more RSF (2.3 [1.7-3.1] vs. 1.8 [1.4-2.5] cm^2^). Hypertensive patients, when compared to normotensive subjects, had a larger RSF depot (2.6 [2.0-3.3] vs. 2.0 [1.4-2.5] cm^2^), even after considering BMI. In combined data, RSF showed a negative correlation with estimated glomerular filtration rate (eGFR) but had no association with systolic or diastolic blood pressure. After weight loss surgery, RSF decreased along with other obesity markers. The magnitude of RSF reduction was greater in patients who experienced hypertension relief compared to those who still had hypertension (-0.68 [-0.74, -0.44] vs. -0.28 [-0.59, 0] cm2, p = 0.009). The accumulation of RSF appears to be linked to the pathogenesis of obesity-related hypertension, and post-surgery, a significant decrease in RSF was observed, correlating with relief from hypertension (31). Similarly, in a study involving dietary-induced weight loss over an 18-month period, a correlation was observed between RSF and baseline eGFR as well as microalbuminuria. After 8 months, RSF decreased by 6.18%, and this reduction was associated with overall body weight loss. The decrease in RSF was also linked to improvements in lipid profiles and blood glucose control (37).

### 4.2 Weight reduction and glomerular filtration rate

Studies, including one by Lin et al., demonstrate that bariatric surgery was associated with eGFR preservation in all obese patients and, particularly, in those with moderate-to-high CKD risks. The study revealed a significant negative correlation was evident between an increased eGFR and a reduced BMI (Spearman’s correlation -0.229, P < 0.001),and the bariatric surgery group had a significantly lower risk of an eGFR decline ≥25% at 12 months [adjusted HR (aHR) 0.47, P = 0.03). After BS, obese patients with hypertension or albuminuria had significantly lower risks of eGFR declines ≥25% (aHR 0.37, P = 0.02 and aHR 0.13, P = 0.0018, respectively) (64). In a prospective cohort study, researchers observed analogous trends. Twenty-five individuals, comprising both obese and non-diabetic subjects, exhibited considerable stability in unadjusted mean glomerular filtration rate (mGFR) and a noteworthy improvement in adjusted mGFR (65). Caloric restriction-based dietary interventions also exhibit positive effects. Individuals with eGFR < 120 mL experience an overall increase in GFR with weight loss, while those with hyperfiltration witness a substantial decline in GFR, indicating a favorable reduction in obesity-associated glomerular hyperfiltration (66–68). Moreover, utilizing advanced imaging techniques, researchers provided compelling evidence that obesity induces structural, metabolic, and hemodynamic changes in the kidneys. Obese subjects showed higher renal volume but lower radiodensity, suggestive of potential water and/or lipid accumulation. Cardiac output and eGFR were increased by approximately 25% in obese individuals. Total renal blood flow was higher in the obese, and FFA uptake was about 50% higher due to elevated circulating FFA levels. Importantly, following weight loss (26 ± 8 kg), these changes in eGFR, total renal blood flow, kidney volume, FFA uptake, and renal density were partially reversed, thereby mitigating the risk of obesity-induced progression of chronic kidney disease (69).

In conclusion, weight reduction offers multifaceted benefits for both diabetes and obesity, including improved metabolic disturbances, reduced adipose tissue deposition, alleviated chronic inflammation, and restored renal structure and function. For patients with obesity and concurrent diabetes, weight reduction interventions are beneficial for renal health, potentially delaying or preventing renal function impairment (70).

**Figure 1:**
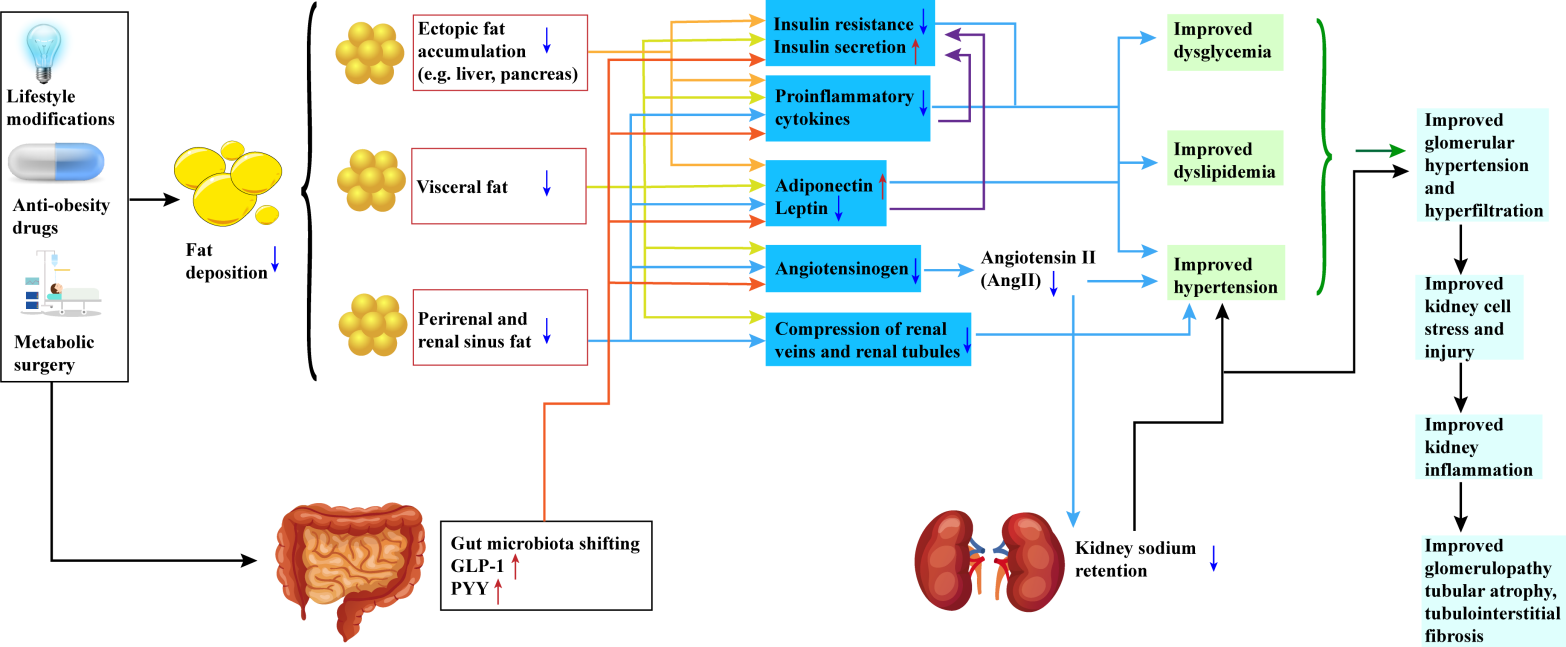
Potential Mechanisms of Weight Loss Intervention in Preserving Renal Function in Obesity and Diabetic Patients. All three approaches—lifestyle modifications, anti-obesity medications, and metabolic surgeries—result in a reduction in patient body weight. This reduction is characterized by a decrease in fat deposition, encompassing visceral fat, renal sinus fat, and ectopic adipose tissue. The reduction of fat in these specific regions promotes the restoration of insulin sensitivity, elevation of peripheral insulin levels, suppression of pro-inflammatory factors, and modulation of adipokine release. Simultaneously, alterations in the release patterns of adipokines are observed, marked by an increase in adiponectin levels and a decrease in leptin levels. Furthermore, the renin-angiotensin-aldosterone system (RAS) is inhibited. Additionally, shifts in gut microbiota composition and increased secretion of hormones such as GLP-1 and PYY contribute to these effects. Collectively, these changes culminate in enhanced regulation of blood glucose, blood lipids, and blood pressure. The inhibition of the RAS system also leads to reduced urinary sodium retention, providing a safeguard for the kidneys. These interventions exert a favorable impact on renal function, including the amelioration of glomerular hyperfiltration, preservation of renal cell integrity, and reduction in renal inflammation. Ultimately, these mechanisms translate into enhancements in kidney structure, including the attenuation of glomerular and renal tubular atrophy, as well as a reduction in interstitial fibrosis. GLP-1(Glucagon-Like Peptide-1); PYY(Peptide YY). Red arrows represent an increase, and blue arrows represent a decrease.

In **Figure 1**, we elaborate on the specific mechanisms illustrating the impact of weight loss intervention on renal function in obese and diabetic patients.

## 5 Perspectives and conclusion

Obesity combined with diabetes poses a significant health challenge globally, with the associated renal issues posing a serious threat to patient health. Weight loss interventions show promising potential in improving renal function, and current research has made some encouraging progress. However, future research directions and key issues still require further exploration to enhance our understanding of this crucial area and ultimately improve patients’ quality of life. Firstly, mechanistic studies will continue to be a focal point. Understanding how weight loss interventions impact renal function through biochemical pathways, and delving into the molecular and cellular mechanisms between obesity, diabetes, and renal diseases, is crucial for developing more targeted intervention strategies. These studies are expected to unveil new therapeutic targets and opportunities for drug development. Secondly, long-term effects and safety assessments will be a critical area for future research. Over time, the long-term impact of weight loss interventions on renal function needs more attention, especially regarding their role in the progression of renal diseases. Personalized and comprehensive interventions represent another important future direction. Different patients have diverse genetic backgrounds, lifestyles, and disease stages, making personalized intervention plans crucial. Integrating drug therapy, lifestyle adjustments, even metabolic surgery to optimize renal function improvement will be a key goal of future research and clinical practice.

In conclusion, the impact of weight loss interventions on renal function in patients with obesity and diabetes is a research area of significant clinical importance. With further research and technological advancements, we can not only better comprehend its mechanisms but also formulate more effective treatment strategies, ultimately offering diabetic and obesity patients improved health and quality of life.

## Author contributions

XG: Conceptualization, Data curation, Methodology, Writing – original draft. XZ: Conceptualization, Methodology, Supervision, Validation, Writing – review & editing. PF: Conceptualization, Methodology, Supervision, Validation, Writing – review & editing.
